# Lotus Leaf-Derived Gradient Hierarchical Porous C/MoS_2_ Morphology Genetic Composites with Wideband and Tunable Electromagnetic Absorption Performance

**DOI:** 10.1007/s40820-020-00568-1

**Published:** 2021-01-04

**Authors:** Fei Pan, Zhicheng Liu, Baiwen Deng, Yanyan Dong, Xiaojie Zhu, Chuang Huang, Wei Lu

**Affiliations:** 1 Shanghai Key Lab. of D &A for Metal-Functional Materials, School of Materials Science & Engineering, Tongji University, Shanghai, 201804 People’s Republic of China; 2 School of Materials Science & Engineering, University of Shanghai for Science and Technology, Shanghai, 200092 People’s Republic of China

**Keywords:** Morphology genetic materials, Lotus leaf, Electromagnetic wave absorption, Gradient hierarchical porous structure, Dielectric sum-quotient model

## Abstract

Inspired by the nature, lotus leaf-derived gradient hierarchical porous C/MoS_2_ morphology genetic composites (GHPCM) were successfully fabricated through an in situ strategy. The biological microstructure of lotus leaf was well preserved after treatment. Different pores with gradient pore sizes ranging from 300 to 5 μm were hierarchically distributed in the composites. In addition, the surface states of lotus leaf resulted in the Janus-like morphologies of MoS_2_. The GHPCM exhibit excellent electromagnetic wave absorption performance, with the minimum reflection loss of − 50.1 dB at a thickness of 2.4 mm and the maximum effective bandwidth of 6.0 GHz at a thickness of 2.2 mm. The outstanding performance could be attributed to the synergy of conductive loss, polarization loss, and impedance matching. In particularly, we provided a brand-new dielectric sum-quotient model to analyze the electromagnetic performance of the non-magnetic material system. It suggests that the specific sum and quotient of permittivity are the key to keep reflection loss below − 10 dB within a certain frequency range. Furthermore, based on the concept of material genetic engineering, the dielectric constant could be taken into account to seek for suitable materials with designable electromagnetic absorption performance.

**Supplementary Information:**

The online version of this article (10.1007/s40820-020-00568-1) contains supplementary material, which is available to authorized users.

## Highlights

- Lotus leaf-derived gradient hierarchical porous C/MoS_2_ morphology genetic composites nanocomposites were fabricated.
- Excellent electromagnetic absorption performance was achieved with *RL*_min_ of − 50.1 dB and EBW of 6.0 GHz.
- A brand-new dielectric sum-quotient model was proposed and corresponded well to the experimental results.

**Supplementary Information:**

The online version of this article (10.1007/s40820-020-00568-1) contains supplementary material, which is available to authorized users.

## Graphical abstract

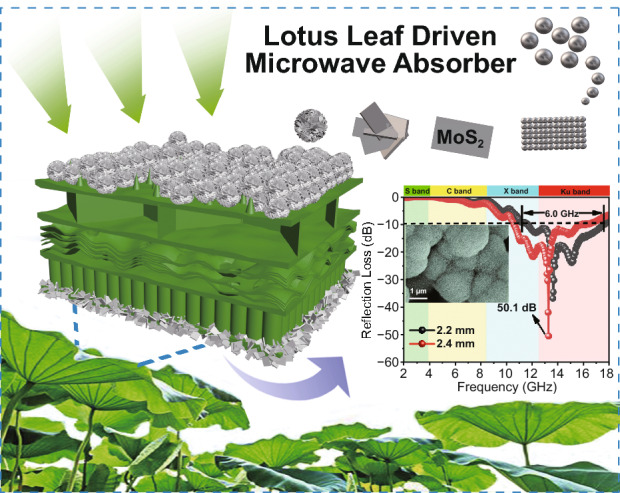

## Introduction

In recent years, with the gradual rise of 5G technology, more and more electronic devices rapidly appear in the world. These new-style products bring convenience to our life, and they also cause electromagnetic radiation problem, threating to our health [1]. In view of this background, it is urgent to develop electromagnetic absorbing or shielding materials in the GHz frequency band to overcome this problem. Electromagnetic wave (EMW) absorber, which composed of dielectric loss component and magnetic loss component, can convert electromagnetic wave into other forms of energy through synergistic action of different loss mechanisms and impedance matching [2]. At present, researches on the properties of EMW absorbers mainly focus on four aspects, including low filling ratio, low matching thickness, wide effective frequency band, and strong reflection loss [3]. From traditional materials such as graphene [4], carbon nanotubes [5], and ferrites [6] to new generation materials such as MOFs [7], aerogel [8], and MXene [9], all have been successfully fabricated and exhibit gratifying EMW absorption performance.

In nature, the morphological structure of organisms has undergone eons of evolution, showing the characteristics of fine structure and functional integration. Based on the enlightenment from this perspective, the concept of morphology genetic materials (MGMs) has been proposed in recent years, focusing on the functionalization of materials while preserving the morphological structure of the organism itself [10]. In past few years, it has been found that the carbon skeleton of plants, animals, or microorganisms obtained by carbonization treatment at high temperature has the potential to be a good EMW absorber [11]. Moreover, introducing some special structural treatment methods in the preparation process can effectively enhance the absorption performance. Hole-creating is a frequently used idea to optimize the EMW absorbing properties of MGMs. The existence of mesoporous hole can not only increase the reflection of EMW inside the material, but also effectively adjust the dielectric according to Maxwell–Garnet model [12]. In the field of EMW absorption materials, potassium hydroxide activation and low temperature sublimation are often used to enrich the pore structure of MGMs. Zhou et al. used fish skin as carbon source to fabricate 3D carbon foams that exhibit a minimum reflection loss (*RL*) of − 33.5 dB at 3 mm with an effective bandwidth of 8.6 GHz (*RL* < − 10 dB). After KOH treatment, the pore structure of fish skin was greatly improved, resulting in the enhanced EMW properties [13]. Liang et al. fabricated natural eggplants-derived silicon carbide aerogels by freeze-drying. The moisture in the eggplant run off at low temperatures to process a three-dimensional porous structure, which increases the specific surface area of the material. The obtained aerogels showed a minimum *RL* of − 43 dB at 2 mm thickness and an effective bandwidth of more than 4 GHz [14]. Although the results of hole-creating engineering are satisfactory, the complicated treatment processes may destroy the intrinsic morphology of the raw material, which is contrary to the original intention of the MGM design. Therefore, it is still challenging to find new biological materials with unique morphology genetic structure in nature and explore the influence of structure on performance.

In the design of EMW absorber, few works were concentrated on the gradient hierarchical porous structure and Janus structure. For the former, because the synthesis process cannot be precisely regulated under the micron and nanometer size, the general material system can hardly obtain the structure of pore classification arrangement with different diameters. According to waveguide theory, when the wavelength is two times higher than the length of the waveguide’s cross section, the microwave would be attenuated inside the waveguide [15]. Different apertures correspond to different wavelengths of EMW, which means that the graded distribution of the pore structure is beneficial to the broadening of bandwidth. Lotus leaf, as one of the most common plants in summer, is composed of interlayer with different pore sizes, which is quite consistent with the construction of hierarchical porous structure. More interestingly, the presence of papillae makes the front side of the lotus leaf appear hydrophobic, distinct from the hydrophilic structure on the back side. This Janus-like structure endows the anisotropy of the lotus leaf due to the asymmetry in the morphology and the difference in surface chemistry [16]. At present, research for preparing Janus particles at the nanoscale has been applied to the absorber. Shi et al. fabricated Fe_3_O_4_/PDA asymmetric Janus nanoparticle via polymerization of dopamine in a Pickering emulsion. The obtaining nanocomposite showed strong *RL* (− 50 dB) and wide bandwidth which covered 70% of whole frequency range [17]. Therefore, the different properties on both sides of lotus leaf provide the possibility to construct Janus structure at the microscale. Unfortunately, there is currently no research on EMW absorbing materials with lotus leaf as carbon source.

In addition to the selection of the MGM, the composite is also an important strategy to improve the EMW absorption performance. Due to the high degree of graphitization, the MGM leads to excess conductivity in the system, making the material approach to the ideal conductor and prevents the EMW from entering the material [18]. On the one hand, the composite strategy can effectively adjust the conductive loss of the MGM because of “throttling” effect; on the other hand, heterogeneous interface and defects are formed in the final binary system, which increases the polarization loss [19]. Molybdenum disulfide (MoS_2_), a transition-metal dichalcogenides with similar structure to graphite, has been widely used in functional materials. The Mo atomic layer is bound between the two S atomic layers by Vander Waals interactions [20]. Under the alternated electromagnetic field, dissipated current in MoS_2_ is formed by the induced electrons shifting along the molecular network, resulting in a good response to EMW [21]. This excellent electromagnetic response and stable physical and chemical properties make MoS_2_ a popular composite choice. So far, MoS_2_ has been combined with ZnO [22], FeS [23], RGO [24], and Fe_3_O_4_ [25] to obtain excellent absorbing performance. However, there is still a lack of report on the composite of MoS_2_ and MGM.

Herein, we successfully prepared lotus leaf-derived GHPCM morphology genetic composites by an in situ method and followed by a carbonization process. A moderate heating rate makes lotus leaf to retain its original morphology after carbonization, including frontal papillae morphology. The hierarchical porous structure with gradient pore sizes and MoS_2_ with Janus morphologies are obtained in the MGM composites. More importantly, good electromagnetic performance was obtained in the MGM composites: a minimum *RL* of about − 50.1 dB at 13.24 GHz with thickness of 2.4 mm and the maximum effective bandwidth of 6.0 GHz from 11.6 to 17.6 GHz at thickness of 2.2 mm, covering the whole Ku band. Moreover, a dielectric sum-quotient model is put forward for the first time to analyze the absorption performance from a computational perspective. This work makes full use of the advantages of MGM and obtain a hierarchical porous morphology via a facile synthesis method, which is hard to achieve from traditional materials. The present results suggest that developing highly efficient EMW absorbing materials from nature will be a renewable, eco-friendly, and feasible way in future.

## Experimental Section

### Materials

Lotus leaf were purchased from Yangqingtang (Jiangsu, China). Thiourea (NH_2_CSNH_2_) and ammonium molybdate tetrahydrate [(NH_4_)_6_Mo_7_O_24_·4H_2_O] were both obtained from Sinopharm Chemical Reagent Co., Ltd., Beijing, China. All chemical reagents are of analytical grade and used were used as received without further purification. Deionized water obtained from a Milli-Q system was used all the time.

### Preparation of GHPCM

**Fig. 1:**
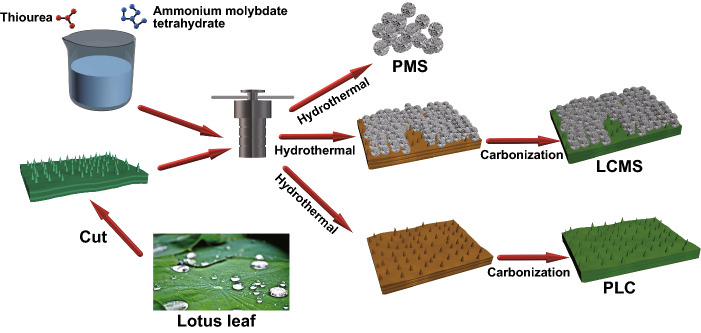
Schematic illustration of the GHPCM formation process

The preparation of GHPCM is illustrated in Fig. 1, and the detail steps are as follows: First, the dried lotus leaves was cut into 2 × 2 cm^2^ square slices and washed with alcohol and deionized water for several times. Then, six pieces of lotus leaves slices, 1 g of thiourea, and 0.2 g ammonium molybdate tetrahydrate were added into 30 mL deionized water, following by vigorous stirring for 0.5 h. The mixed solution was transferred into a tetrafluoroethylene-lined stainless steel autoclave heated at 200 °C for 24 h to obtain hydrothermal C/MoS_2_. The resulting black slices were washed with deionized water via a suction filtration process, drying overnight at 60 °C. Finally, the dried powder was heated to X °C (X = 600, 700, 800) and held it for 1 h in Ar atmosphere at a rate of 1 °C min^−1^. After cooling to room temperature with furnace, the final GHPCM were denoted as LCMS-600, LCMS-700, and LCMS-800, respectively.

### Preparation of Pure MoS_2_ and Pure Lotus Leaf Carbon

Pure MoS_2_ and lotus leaf carbon was also fabricated as a control group in order to study the influence of components on the absorption performance. 1 g of thiourea and 0.2 g ammonium molybdate tetrahydrate were added into 30 mL deionized water, following by vigorous stirring for 0.5 h. The mixed solution was transferred into a tetrafluoroethylene-lined stainless steel autoclave heated at 200 °C for 24 h to obtain pure MoS_2_, which was denoted as PMS. After the same hydrothermal treatment without adding thiourea and ammonium molybdate tetrahydrate, lotus leaves slices were heated to 700 °C and held it for 1 h in Ar atmosphere at a rate of 1 °C min^−1^. The final black Lotus leaves were denoted as PLC-700.

### Characterizations

The X-ray diffraction (XRD) studies were carried out on DX-2700 X-ray diffractometer using Cu-Ka radiation (*λ* = 1.54 Å). The morphology was characterized by scanning electron microscopy (SEM) and transmission electron microscopy (TEM). The thermogravimetry (TG) and differential scanning calorimetry (DSC) curve was characterized using a Netzsch TG thermal gravimetric analyzer in a N_2_ atmosphere 30–800 °C. The differential thermal analysis (DTA) curve was characterized using a SDT Q600 in a N_2_ atmosphere 30–800 °C. ASAP2460 instrument was used to characterize the nitrogen adsorption and desorption isotherms, and the specific surface area was measured by the Langmuir method. Pore size distribution was deduced from the absorption isotherms by density functional theory. Raman spectrum was characterized by a cryogenic matrix isolated Raman spectroscopic system using a 532 nm laser. X-ray photoelectron spectroscopy (XPS) was performed on a Thermo Scientific K-Alpha spectrometer. Electrical conductivities were detected by utilizing a standard four-probe station (HPS2524). Based on coaxial line theory, the EMW parameters of samples were measured over the 2–18 GHz range in a vector network analyzer (VNA, 3672B-S, Ceyear). Samples were prepared by uniformly mixing the products with paraffin at a mass fraction of 40 wt% and then compacted into a columnar ring of 7.00 mm outer diameter and 3.04 mm inner diameter. The final complex permeability and complex permittivity were determined from the experimental scattering parameters through the standard Nicolson-Ross and Weir theoretical calculations.

## Results and Discussion

### Characterization of GHPCM

**Fig. 2:**
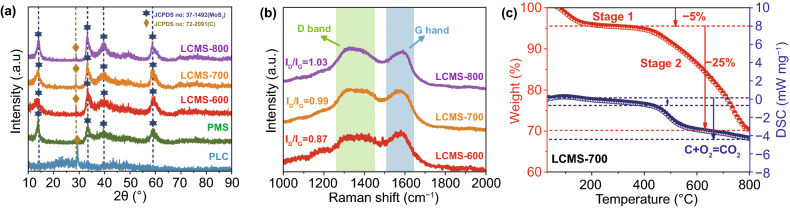
**a** XRD patterns of samples. **b** Raman spectroscopy of GHPCM. **c** TG and DCS curve of the of GHPCM

The crystallographic structures and phase contents of the as-synthesized samples were investigated by XRD analysis shown in Fig. 2a. As for PMS, the peaks at 14.3°, 32.7°, 39.5°, and 60.1° are assigned to (002), (100), (103), and (008) crystal planes of 2H–MoS_2_ (JCPDS No. 37–1492) with hexagonal structure [24]. As for PLC, the peaks at 29.5° and 42.2° are assigned to (110) and (200) crystal planes of carbon (JCPDS No. 72–2091). As labeled in Fig. 2a, it can also be observed in the composites LCMS-600, LCMS-700, and LCMS-800, which infers the existence of crystalline phases for both 2H–MoS_2_ and carbon as well as the successful synthesis of the composites. According to Scherrer equation: *D* = k*λ*/*B*cos *θ*, where *D* is the interplanar distance, k is the Scherrer constant, *λ* is the X-ray wavelength, *B* is the line broadening at half the maximum intensity, and *θ* is the scattering angle, the average size of MoS_2_ crystalline particles is about 2.84 nm. To further explore the composition and molecular structure of the samples fabricated at different temperatures, the results of Raman spectroscopy are shown in Figs. 2b and S1a. Three peaks at about 280, 360, and 408 cm^−1^, originating from E_1g_, E_2g_^1^, and A_1g_ Raman vibrational modes of 2H–MoS_2_, respectively, are detected in the composites LCMS-600, LCMS-700, and LCMS-800 [26]. In addition, two distinct peaks located at 1360 and 1580 cm^−1^ are the D and G bands of the carbon phase, respectively [27]. Generally speaking, the D band is correlated with the disorder or structure defects in the *sp*^2^-hybridized carbon atoms or amorphous carbon deposits, while the G band is associated with the in-plane vibrations of *sp*^2^ atoms in a 2D hexagonal graphitic lattice [28]. The intensity ratio of two band (*I*_D_/*I*_G_) represents the disorder degree of material to some extent. Herein, *I*_D_/*I*_G_ is calculated to be 0.87, 0.99, and 1.03 of LCMS-600, LCMS-700 and LCMS-800, respectively, indicating the enhanced disordering with the increased carbonization temperatures. The weight loss of the LCMS-700 during heat treatment is also detected by using TG- DSC and TG–DTA analysis under N_2_ atmosphere from 30 to 800 °C (Fig. 2c and S1b). The first-stage weight loss of about 5% appears from 50 to 200 °C and is probably resulted from the removal of surface adsorbed water or hydroxyls. And the second-stage weight loss of about 25% occurred from 200 to 800 °C and is mainly attributed to the decomposition of organics in lotus leaves. Moreover, the two weight loss processes accurately correspond to the two endothermic processes on the DSC curve. Meanwhile, DTA curve also shows two endothermic signals.

**Fig. 3:**
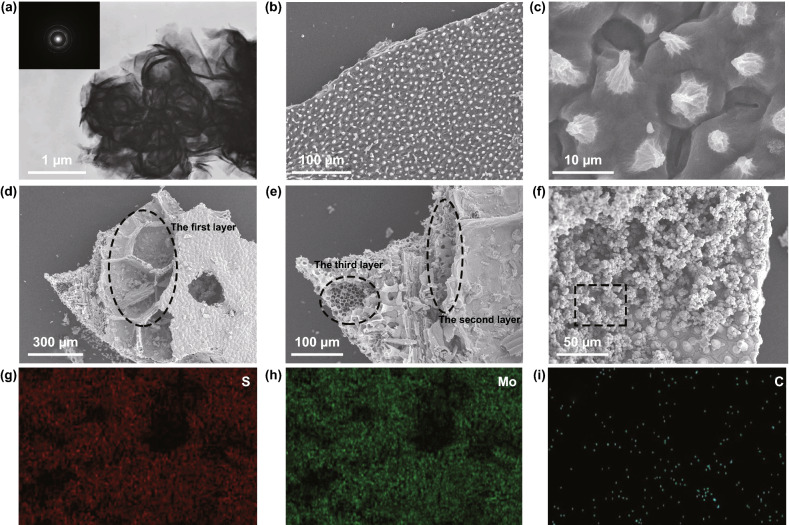
**a** TEM images of PMS. **b**–**c** SEM images of PLC**. d**–**f** SEM images of LCMS-700. **g**–**i** EDS of S, Mo, and C elements of LCMS-700

Figure 3 exhibits the SEM and TEM images of the as-synthesized samples. Figure 3a shows the TEM image of the PMS which reveals a typical flower-like architecture. The size of the flower formed by the assembly of the sheets is about 1 μm. The selected area electron diffraction (SAED) patterns in the insert exhibits distinct polycrystalline fringes. Therefore, it is inferred that under hydrothermal conditions, MoS_2_ grains are stacked to form MoS_2_ sheets, and thousands of sheets are stacked to form the flower-like morphology subsequently. As shown in Fig. 3b, c, circular cone-like papillae are neatly distributed on the surface of lotus leaf with micron-sized holes surrounding. This unique structure on the front side was well maintained during the hydrothermal and carbonization processes and increases the contact angle when lotus leaf comes into contact with water droplets, resulting in hydrophobic characteristics of surface. In addition, the GHPCM are clearly visible in the SEM images shown in Fig. 3d–f. The results indicate that carbonizing at 1 °C min^−1^ is a suitable condition to maintain the original morphology of MGM. From Fig. 3d, e, a broken GHPCM is selected to facilitate the analysis of the internal hierarchical structure of lotus leaf. We simply divide the lotus leaf into three layers, which are named as huge-macropore layer, loose-packed macropores layer, and close-packed macropores layer, respectively. In the macropore layer, a honeycomb arrangement of square cavities with apertures above 300 μm is discovered. The original bound water or organics in the cavity is sufficiently volatilized during carbonization. Pore sizes of loose-packed macropores layer and close-packed macropores layer are similar, which are between 5 and 10 μm. The difference between them is the spacing between the pores. As for loose-packed macropores layer, pore and pore are connected by a carbon layer of 15 μm, while pores in close-packed macropores layer are directly in contact with each other. Thus, the lotus leaf formed with triple-layer connection has a hierarchical structure with multiple types of pores, which is rarely observed in other carbon-based materials. As shown in Fig. 3f, it is clear that hundreds of MoS_2_ flowers lay on the surface of the lotus leaf. The relevant elemental distribution of the GHPCMs is shown in Fig. 3g–i and details are presented in supplementary material (Fig. S2 and Table S1). According to the results, the distribution of S and Mo elements are almost the same, and the corresponding atomic ratio is also close to 2:1, which further proves the successful preparation of MoS_2_. Besides, C content is quite low in mapping because stacked MoS_2_ on the C surface makes the rays impenetrable. To study the specific surface area and distribution of pore size, the N_2_ adsorption–desorption isotherms and pore diameter of LCMS-700 are displayed in Fig. S1c. It can be seen that LCMS-700 demonstrates a representative type IV isotherm with a distinct hysteresis loop at the *P*/*P*_0_ range of 0.4–1.0, which manifest the presence of mesoporous. Moreover, the result of Barrett–Joyner–Halenda (BJH) indicates that the average pore diameter of LCMS-700 mainly focuses at 2.4 and 20.7 nm. The mesoporous structure not only makes composites which possess specific surface area of 155.65 m^2^ g^−1^, but also work together with the other pores shown by the SEM results to provide more paths for the reflection attenuation of EMW.

**Fig. 4:**
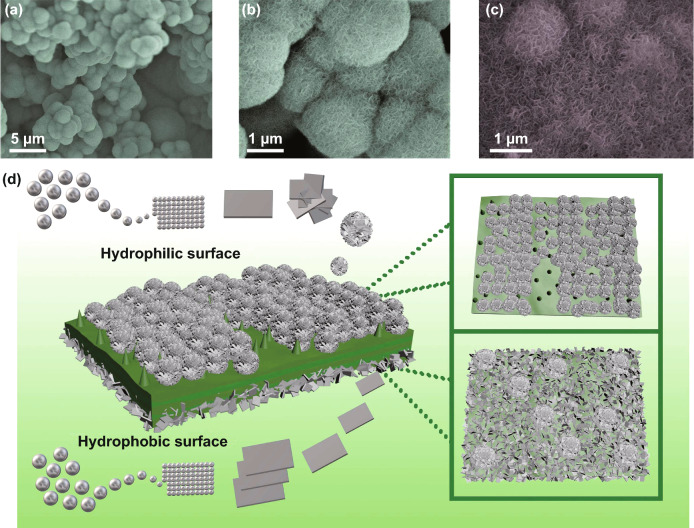
**a**–**b** SEM images of front side of LCMS-700. **c** SEM images of back side of LCMS-700. **d** Schematic illustration of the Janus-like structure formation process

From Fig. 4a–c and S4a–c, visible distinction in MoS_2_ growth is displayed on two sides of lotus leaf. On the front side of the lotus leaf, MoS_2_ all existed in flower-like morphology. While on the back side, most part of MoS_2_ is flaked on the surface, and the remaining part exists in the form of hemisphere. The extraordinary growth mode of MoS_2_ allows GHPCM to show an anisotropic structure similar to Janus on the microscopic level. In view of the morphology and characteristics of two sides of lotus leaf, the specific formation mechanism we speculated is shown in Fig. 4d. At the initial stage of hydrothermal process, MoS_2_ sheets come into being first in the solvent. Due to the hydrophobicity of the front side and the hydrophilicity of the back side, the water solvent is more likely contacted with the back side, which makes the back side first covered with MoS_2_ sheets. Furthermore, the uneven surface of the front side which covers with papillae also limits the growth of MoS_2_ sheets. As the reaction progresses, the high temperature gradually destroys the structure of the hydrophobic surface. At the same time, MoS_2_ sheets in the solvent are assembled into flower-shaped MoS_2_, which are dispersed on the front side with the morphology presented in Fig. 4a. And the back side is against the flower-shaped MoS_2_ growth owing to the increased surface energy caused by previous MoS_2_ sheets.

**Fig. 5:**
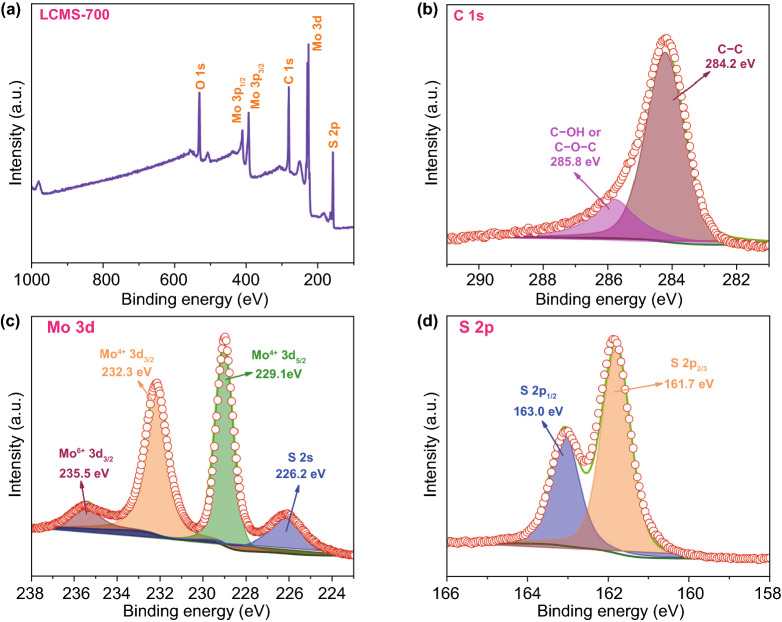
**a** XPS survey scan spectra. XPS core-level spectra of **b** C 1 s, **c** Mo 3d, and **d** S 2p of the LCMS-700

The element analysis of GHPCM has been carried out by XPS to analyze the information on the surface electronic state and the composition of the samples. The XPS survey scan spectrum of the GHPCM in Fig. 5a indicated that the LCMS-700 consisted of element C, O, Mo, and S. The core-level spectra of C, Mo, and S are shown in Fig. 5b–d. As shown in Fig. 5b, the C 1s spectrum consisted of two peaks at ~ 285.8 and ~ 284.2 eV, which were related to C–O–C and C–C, respectively [25]. For Fig. 5c, the high-resolution spectrum of Mo could be deconvoluted into three peaks. The Mo^4+^ 3d_3/2_ (232.3 eV) and Mo^4+^ 3d_5/2_ (229.1 eV) peaks belong to the semiconducting 2H-phase MoS_2_. In the meantime, a satellite peak appears at 235.5 eV, which perhaps owe to the formation of Mo–S–C and Mo–C at the interface between MoS_2_ and carbon [20]. In addition, the peak at 226.2 eV is ascribed to S 2s [23]. The coexistence of two peaks is well demonstrated in the high-resolution spectra of S 2p from Fig. 5d, where the peaks at ~ 161.7 and ~ 163.0 eV were consistent with the S^2−^ 2p_3/2_ and S^2−^ 2p_1/2_ orbitals, respectively [29]. These data further demonstrated that both carbon and MoS_2_ were successfully combined, which can ultimately trigger intensive polarization loss to consume EMW energy.

### Microwave Absorbing Property of GHPCM

**Fig. 6:**
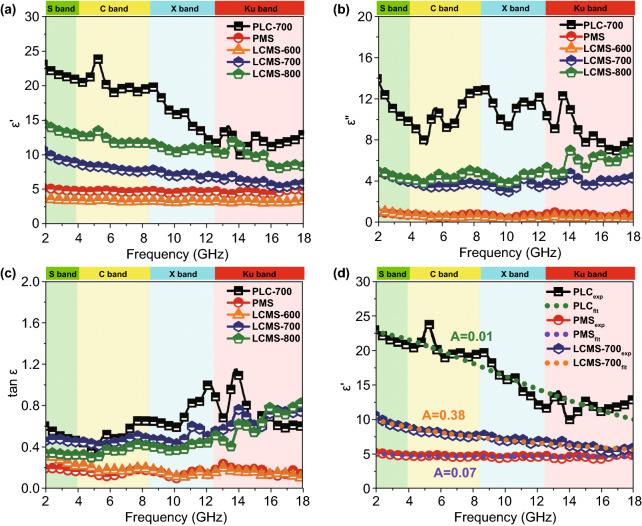
**a**–**b** real and imaginary parts of permittivity. **c** Dielectric loss tangents. **d** Real part of permittivity of measured and empirical formula fitted of PLC, PMS, and LCMS-700

In general, relative complex permittivity (*ε*_r_ = *ε*′ − *jε*″) and permeability (*μ*_r_ = *μ*′ − *jμ*″) are utilized to investigate the EMW properties of materials. The permeability (Fig. S4) is ignored due to the absence of a magnetic component, and the permittivity of samples is shown in Fig. 6a–c. In common, on the basis of electromagnetic theory, the real parts *ε*′, *μ*′ and imaginary parts *ε*′′, *μ*″ of permittivity and permeability represent the storage and the dissipation capacity of EMW energy, respectively [30]. It can be clearly seen that the PLC-700 exhibits the highest *ε*′ and *ε*″ value from 22.9 to 13.1 and 13.8 to 7.8, respectively. On the contrary, the PMS displays the lowest *ε*′ and *ε*″ value which fluctuates around 5 and 1. Once the MoS_2_ is grown in situ on the surface of the lotus leaf, *ε*′ and *ε*″ significantly decrease comparing to that of the PLC and show an upward trend with the increased carbonization temperatures. In detail, the *ε*′ values of LCMS-600, LCMS-700, and LCMS-800 decrease from 3.7 to 3.4, 10.6 to 5.7, and 14.5 to 8.2, respectively. Similarly, the *ε*″ values of three samples gradually vary from 1.3 to 0.4, 4.9 to 4.3, and 5.0 to 6.7, respectively. On the basis of the dielectric loss theory, the conductive loss and polarization loss are the main factors affecting the permittivity in 2–18 GHz range [31]. The conductance of materials can be simply expressed as a free electron formula (*ε*″ ≈ *σ*/2π*fε*_0_), indicating that conductivity is directly proportional to the *ε*″ [32]. And the polarization loss can be divided into interfacial polarization and dipole polarization [33].

To better analyze the dielectric loss, the samples were divided into two groups according to different components and carbonization temperatures. Conductive loss plays a dominated role in the component group, which covers samples of PLC-700, PMS and LCMS-700. There are three modes of electrons transport in the conductive network, including migrating, hopping, and tunneling. The unique atomic structure and hybridization state of carbon materials give them excellent current conduction capability [3]. So, PLC has higher conductive loss than pure MoS_2_ sample, resulting in a higher *ε*″ value. When the low conductivity MoS_2_ phases were grown on the surface of PLC, the conductivity of the originally flowing electrons is greatly reduced in the process of passing through these “obstacles.” As a result, the excess conductivity of MGM is tuned and a moderate permittivity is obtained at the same time. In addition to the conductive loss, the formation of interfacial polarization also plays a part of role. When MoS_2_ is added, the potential difference between the carbon and MoS_2_ causes charge accumulation at the interface under electromagnetic field and intensifies the polarization relaxation, which is helpful for dielectric loss [34]. To establish the relationship between interface structure and the permittivity, a Debye relaxation correction formula is described as Eqs. 1 and 2 [35]:

1 $$\varepsilon_{\text{r}} = \varepsilon_{{{\text{r}}\infty }} + \frac{{\varepsilon_{{{\text{rs}} }} - \varepsilon_{{{\text{r}}\infty }} }}{{1 + (i\omega \tau )^{1 - A} }}\quad (0 < A < 1)$$

2 $$\varepsilon_{\text{r}}^{{\prime }} = \varepsilon_{{{\text{r}}\infty }} + (\varepsilon_{\text{rs}} - \varepsilon_{{{\text{r}}\infty }} )\frac{{1 + (\omega \tau )^{(1 - A)} \sin \frac{\pi A}{2}}}{{1 + 2(\omega \tau )^{1 - A} \sin \frac{\pi A}{2} + (\omega \tau )^{2(1 - A)} }}$$

where *A* is defined as the interface factor, *ω* is the angular frequency, *τ* is viewed as the relaxation time, *ε*_rs_ and *ε*_r∞_ are the static dielectric constant and limiting dielectric constant, respectively. According to previous reports, the higher value of *A*, the more interface polarization there are [35]. Figure 6d reveals the fitting curves of real part of permittivity, and the corresponding A values of PLC, PMS, and LCMS-700 are 0.01, 0.07, and 0.38, respectively. The increased A value of LCMS-700 represents that the combination MoS_2_ and carbon effectively improves the interface polarization. Although LCMS-700 is rich in interfacial polarization, it should be noted that the permittivity of LCMS-700 is still lower than that of PLC-700 because the proportion of interfacial polarization is much lower than the conductivity loss. Unlike the component group, dipole polarization is dominant in the carbonization temperature group, covering samples of LCMS-600, LCMS-700, and LCMS-800. Defect and functional groups on the surface of materials usually act as polarization center to bring about polarization relaxation under the impact of EMW field [36]. The intrinsic dipole moments of these centers cannot sum to zero, resulting in the dipole polarization. Once increasing the frequency of EMW field, the polarizability fails to maintain the original state, enhancing the dielectric loss [37]. According to the previous Raman results, the disordering degree of the GHPCM increases with the increased carbonization temperatures, suggesting that the material has more surface defects at higher carbonizing temperature. The dipole polarization dominated by these defects makes the permittivity of sample LCMS-600, LCMS-700, and LCMS-800 proportional to the temperature. A Cole–Cole semicircle model is used to better explain the polarization loss occurred in the EMW attenuation, which is described as Eq. 3 [38]:

3 $$\left( {\varepsilon^{\prime } - \frac{{\varepsilon_{{{\text{s}} }} + \varepsilon_{\infty } }}{2}} \right) + (\varepsilon^{\prime \prime } )^{2} = \left( {\frac{{\varepsilon_{{{\text{s}} }} - \varepsilon_{\infty } }}{2}} \right)^{2}$$

Each semicircle in the *ε*′–*ε*″ curves stands for a polarization relaxation process (as shown in Fig. S5) [39]. The number of semicircles in the figure increases gradually with the increasing carbonization temperature, which represents that high temperature is conducive to the formation of dipole polarization process. To illustrate this point more intuitively, we quantitatively differentiate the conductance loss ($\varepsilon_{\text{s}}^{{\prime \prime }}$) and polarization loss ($\varepsilon_{\text{p}}^{{\prime \prime }}$) from the imaginary part of the permittivity, according to Eq. 4 [40]:

4 $$\varepsilon^{{\prime \prime }} (\omega ) = \varepsilon_{\text{p}}^{{\prime \prime }} + \varepsilon_{\text{c}}^{{\prime \prime }} = (\varepsilon_{\text{s}} - \varepsilon_{\infty } )\frac{\omega \tau }{{1 + \omega^{2} \tau^{2} }} + \frac{\sigma }{{\varepsilon_{0} \omega }}$$

where *ε*_s_, *ε*_∞_, *σ*, and *τ* represent the static dielectric constant, the dielectric constant at infinite frequency, electrical conductivity, and the polarization relaxation time, respectively. Electrical conductivities were detected by utilizing a standard four-probe station (Fig. S6a). Figure S6b, c illustrates the contributions of $\varepsilon_{\text{c}}^{{\prime \prime }}$ and $\varepsilon_{\text{p}}^{{\prime \prime }}$ of samples LCMS-600, LCMS-700, and LCMS-800. In most frequency range, the contribution of $\varepsilon_{\text{p}}^{{\prime \prime }}$ is greater than that of $\varepsilon_{\text{c}}^{{\prime \prime }}$ and increases with the increase in carbonization temperature, which is consistent with the Cole–Cole circle and the Raman results.

**Fig. 7:**
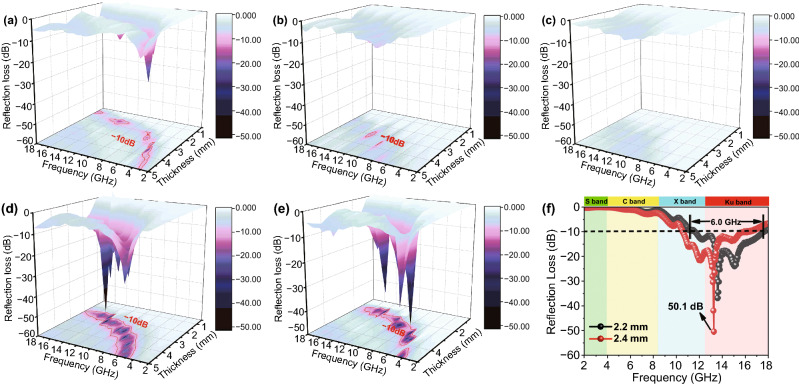
3D reflection loss of the samples with different thicknesses (0.5–5.0 mm) from 2.0 to 18.0 GHz: **a** PLC, **b** PMS, **c** LCMS-600, **d** LCMS-700, and **e** LCMS-800. **f** Reflection loss of LCMS-700 from 2.0 to 18.0 GHz at 2.2 and 2.4 mm

**Fig. 8:**
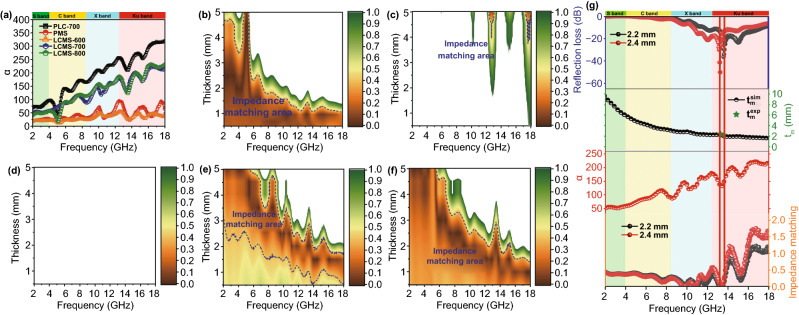
**a** Attenuation constant of samples. Calculated delta value maps: **b** PLC, **c** PMS, **d** LCMS-600, **e** LCMS-700, **f** LCMS-800. **g** Frequency dependent attenuation constant, reflection loss, delta value, and the quarter wavelength thickness of LCMS-700

For further evaluating the EMW absorbing of the composites, the *RL* values at 0.5–5.0 mm thickness in the frequency range of 2–18 GHz are evaluated on the basis of transmission and Debye theory, which can be depicted as Eqs. 5 and 6 [41]:

5 $$Z_{\text{in}} = Z_{0} \left( {\frac{{\mu_{\text{r}} }}{{\varepsilon_{\text{r}} }}} \right)^{1/2} \tanh [j(2\pi fd/c)(\varepsilon_{\text{r}} \mu_{\text{r}} )^{1/2} ]$$

6 $$RL = 20\log \left| {(Z_{\text{in}} - Z_{0} )/(Z_{\text{in}} + Z_{0} )} \right|$$

where *Z*_0_ stands for the impedance of free space, *Z*_in_ is the input impedance of the absorber, *d* is the thickness of the absorber, and c represents the velocity of light. Figure 7a–e displays the 3D *RL* curves of the samples at different thickness with 40% filling ratio. Generally speaking, the minimum *RL* value lower than − 10 dB is often considered as a suitable absorber for practical applications due to 90% of the EMW energy can be attenuated in this situation. From Fig. 7a, b, we can see that the minimum *RL* value of the PLC-700 is up to − 27.2 dB at 4.88 GHz when the thickness is 3.5 mm. And only a negligible part of the effective absorption region indicates the poor absorption performance of the PMS sample. From Fig. 7d–f, it can be observed that LCMS-700 and LCMS-800 show an improved EMW absorption performance because of the tunable conductive loss and enhanced polarization loss. The minimum *RL* value of LCMS-700 reaches − 50.1 dB at 13.24 GHz and the effective bandwidth is 5.80 GHz (from 10.52 to 16.32 GHz) with a thickness of only 2.4 mm. When the thickness reduces to 2.2 mm, the effective bandwidth is up to 6.04 GHz from 11.52 to 17.56 GHz, almost covering whole Ku band. If the carbonization temperature goes up to 800 °C, LCMS-800 has a minimum *RL* value of − 44.2 dB and the corresponding effective bandwidth exceeding − 10 dB is 4.1 GHz. Both the absorption intensity and effective bandwidth are lower than that of the LCMS-700 (Fig. S7). Due to the low carbonization temperature, LCMS-600 shows poor absorbing performance shown in Fig. 7c. Combined with previous analysis of EMW parameters, we speculate that the excessive high or excessive low permittivity count against getting good EMW absorption. To better illustrate this point, the *RL* values are codetermined by the attenuation constant and impedance matching [42]. The attenuation coefficient mainly measures the ability of EMW entering the material to be consumed by dielectric loss, as mentioned earlier. While the impedance matching refers to capacity that EMW can enter the material rather than being reflected on the surface. The attenuation coefficient (*α*) values of samples are calculated as Eq. 7, and relevant curves are displayed in Fig. 8a [43].

7 $$\alpha = \frac{\sqrt 2 }{c}\pi f\sqrt {\left( {\varepsilon^{{\prime \prime }} \mu^{{\prime \prime }} - \varepsilon^{{\prime }} \mu^{{\prime }} } \right) + \sqrt {(\varepsilon^{{\prime \prime }} \mu^{{\prime \prime }} - \varepsilon^{{\prime }} \mu^{{\prime }} )^{2} - (\varepsilon^{{\prime \prime }} \mu^{{\prime }} + \varepsilon^{{\prime }} \mu^{{\prime \prime }} )^{2} } }$$

It can be found that the *α* of PLC-700 exhibits the highest value than that of others, which indicates that the PLC-700 possess the strongest attenuation ability. As the carbonization temperature increases from 600 to 800 °C, values of *α* increase from 20, 50, and 41 to 40, 218, and 233 for LCMS-600, LCMS-700, and LCMS-800 within 2–18 GHz, respectively, sharing the similar variation with permittivity. Figure 8b–f reveals a delta (Δ) model to evaluate the impedance matching of the absorbers and the relevant calculation formula is shown as Eq. 8 [44]:

8 $$\left| \Delta \right| = \left| {\sinh^{2} (Kfd) - M} \right|$$

where *M* and *K* parameter are associated with real part and imaginary part of *ε*_r_ and *μ*_r_. The delta value between 0.4 and 0 suggests a satisfactory degree of impedance matching [45]. Clearly, the terrible impedance matching degree (Fig. 8c, d) for the LMS and LCMS-600 sample account for its poor EMW absorption performance, which should be attributed to the low permittivity. Although the overall impedance matching ability of PLC-700 is quite nice, the low Δ value region is mainly concentrated at low frequencies, where the attenuation coefficient is low. In this case, it is hard to get an appropriate absorption performance in the corresponding frequency band. Therefore, LCMS-700 and LCMS-800 with good impedance matching ability in the corresponding frequency band of high attenuation coefficient will naturally show outstanding absorbing performance. Figure 8g shows the corresponding relationship between *RL*, attenuation coefficient and impedance matching of LCMS-700 at 2.2 and 2.4 mm. The impedance matching is close to 0 at the lowest frequency of *RL* and the attenuation coefficient remains at a high value, which is consistent with the above analysis. Moreover, theoretical and experimental differences in matching thickness and frequency are also verified by the 1/4 wavelength formula: $t_{m} = n\lambda /4 = nc/(4f_{m} \sqrt {\left| {\varepsilon_{\text{r}} } \right|\left| {\mu_{\text{r}} } \right|} )$, where n is positive odd number [46]. If the frequency and thickness at the lowest *RL* value compound this model, the reflected EMW are totally offset at the absorber–air interface due to the 180° phase difference between the incident and reflected EMW in the absorbent [47]. There is no doubt that the LCMS-700 is completely consistent with this model (Fig. S8). In addition, we also tested the absorbing performance of LCMS-700 at different temperature rising rates, as shown in Fig. S9. The EMW parameters of the materials show inconspicuous variation at different heating rates. With the increase in the heating rate, the reflection loss and effective band width of LCMS-700 decrease. Therefore, we consider that 1 °C/min is the most appropriate heating rate to obtain a high-performance absorbing material.

**Fig. 9:**
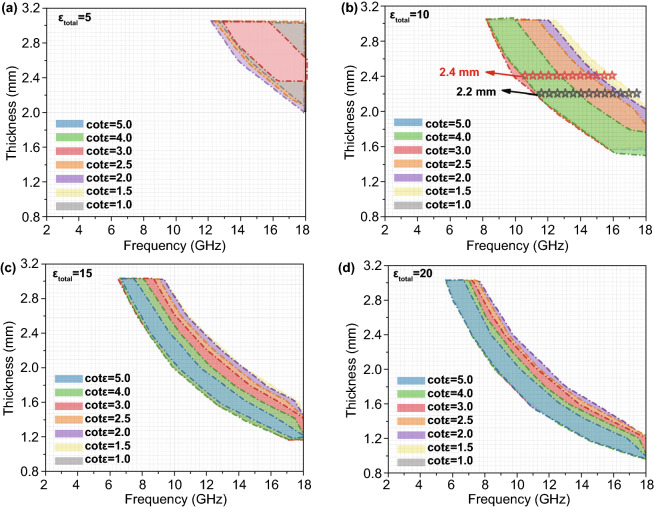
Dielectric sum-quotient model with cotε from 1.0 to 5.0: **a** *ε*_total_ = 5, **b** *ε*_total_ = 10, **c** *ε*_total_ = 15, and **d** *ε*_total_ = 20

In recent years, few mathematical models and simulations have been applied in the analysis of absorbing materials to judge the consistency between the experimental results and theoretical results [48]. In this work, we set up a dielectric sum-quotient model for the first time from the perspective of mathematical calculation to assist the analysis of the cause of the wide bandwidth of the LCMS-700. In allusion to non-magnetic system, we assume that the real and imaginary parts of the permeability over full frequency band are 1 and 0, respectively. By adjusting the numbers of the real and imaginary parts of the permittivity, it can be observed that the final effective *RL* value (*RL* < −10) is influenced by the sum and quotient of two parts. Subsequently, *ε*_total_ is defined as the sum of the real and imaginary parts of the permittivity, and cot *ε* is the quotient of the real and imaginary parts. The relationships between *ε*_total_, cot *ε*, thickness, frequency, and *RL* are established and expressed in Fig. 9a–d, and the relevant data are shown in the supplementary material (Table S2–S5). It can be concluded from the figures that with the increase of ε_total_, the frequency band where the effective *RL* value (*RL *< −10) gradually moves to the low frequency and the effective *RL* value starts to appear at lower thickness. However, the effective *RL* band at the same thickness and cot *ε* are reduced. Combined with the electromagnetic parameters of LCMS-700, *ε*_total_ is close to 10 and cot *ε* is close to 2 at the frequency where *RL* is less than − 10. By matching this value to Fig. 9b, the effective frequency band of LCMS-700 is basically matched with the calculated results. The dielectric sum-quotient model not only verifies the authenticity of the experimental results, but also explains the reason why the material has a wide effective band from the perspective of calculation. In addition, this model will shed light on the designing consideration of the non-magnetic EMW absorbing material systems with different frequency bands and thickness in the future.

**Fig. 10:**
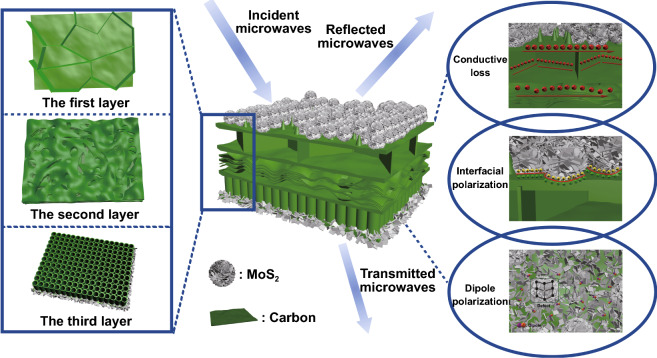
Schematic illustration of the EMW mechanism in the LCMS-700

**Fig. 11:**
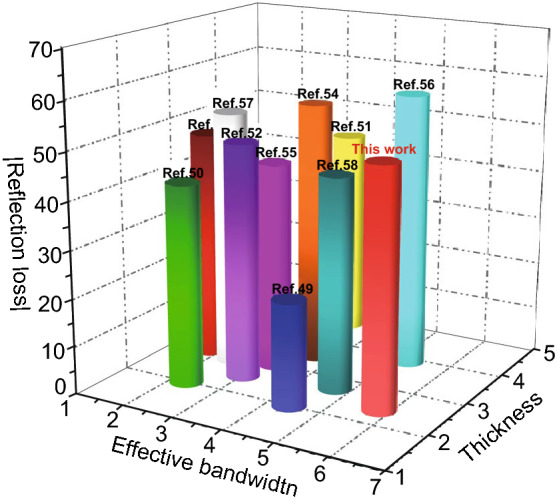
EMW absorption performance of MGM-based materials

Finally, the mechanism of the excellent EMW absorption performance of LCMS-700 is shown schematically in Fig. 10. First of all, the interior hierarchical structures of lotus leaf can be regarded as the result of three layers, including huge-macropore layer, loose-packed macropores layer, and close-packed macropores layer. Due to porous structure of each layer, the EMW entering the material will be reflected back and attenuated in the pores with different sizes. Second, electrons in carbon could absorb EMW energy to migrate in surface/interlayer channels and then convert energy by colliding with the lattice. Moreover, below the percolation threshold, the samples are dispersed in paraffin to form a network of conductance. More electrons hop between different layers of carbon and network conductivity enhances, converting more EMW energy to heat energy. Third, capacitor-like structures would be generated at the interface of heterogeneous interface, which is also named as interfacial polarization. The addition of MoS_2_ introduces the interfacial polarization and modifies the dielectric constant of the samples, so that the impedance matching under high attenuation coefficient is improved. Fourth, numbers of functional groups and defects lead to the asymmetric charge distributions, resulting in the formation of dipoles. Increasing the concentration of dipole polarization makes LCMS-700 suffering more polarization relaxation process, which is beneficial to dielectric loss of the material. In the end, we compared the |*RL*|, effective bandwidth versus thickness for the MGM-based absorption materials published in the recently literature, as shown in Fig. 11 (details in Table S2) [49–58]. It clearly suggests that the outstanding EMW absorption performance of the lotus leaf-derived GHPCM morphology genetic composites (LCMS-700) makes it a potential candidate for practical application in EMW field.

## Conclusion

In this study, morphology genetic C/MoS_2_ composites with gradient hierarchical porous structure were successfully fabricated by a facile in situ method. The morphological structures and the EMW absorption properties of the synthesized GHPCM samples were systematically studied. The obtained morphology genetic C/MoS_2_ composites have a variety of pore structures, giving full play to the peculiar and exquisite features of the morphology of the MGM. Besides, the papillae and hydrophilic–hydrophobic properties of lotus leaf resulted in the Janus microstructure of flower-like and sheet-like MoS_2_. The EMW absorption properties of the GHPCM could be conveniently tuned by the carbonization temperature. Remarkably, LCMS-700 sample achieved a strong reflection loss value of − 50.1 dB at the thickness of 2.4 mm and reaches an effective bandwidth of 6.0 GHz at a relatively thin thickness of 2.2 mm. The multistage pore structure in lotus leaf provides more channels for the reflection and attenuation of EMW. And the introduction of MoS_2_ not only enhances the interfacial polarization but also regulates the excessive dielectric of lotus leaf, which optimizes the impedance matching of composite. Particularly, a dielectric sum-quotient model is put forward based on the mathematical calculations to further verify the experimental results. This investigation provides a new paradigm for the development of pure dielectric loss EMW absorbing materials by taking advantages of the morphology genetic materials and sheds light on the designing consideration of the non-magnetic EMW absorbing material systems in the future.

## Supplementary Information

Below is the link to the electronic supplementary material.

Raman, elemental mappings, permeability, Cole–Cole curves, reflection loss and dielectric sum-quotient model of this work (PDF 824 kb)
