# Supplementary material for: Lotus Leaf-Derived Gradient Hierarchical Porous C/MoS2 Morphology Genetic Composites with Wideband and Tunable Electromagnetic Absorption Performance
Source: Nanomicro Lett. 2021 Jan 4;13:43. doi: 10.1007/s40820-020-00568-1 (PMC8187516; doi:10.1007/s40820-020-00568-1)
Supplement: Supplementary file 1 — Raman, elemental mappings, permeability, Cole–Cole curves, reflection loss and dielectric sum-quotient model of this work (PDF 824 kb) [file 40820_2020_568_MOESM1_ESM.pdf]

Supporting Information for

Lotus Leaf-Derived Gradient Hierarchical Porous C/MoS_2 Morphology Genetic Composites with Wideband and Tunable Electromagnetic Absorption Performance

Fei Pan[1], Zhicheng Liu[1], Baiwen Deng[1], Yanyan Dong[1], Xiaojie Zhu[1], Chuang Huang[2], Wei Lu[1,]*

[1]Shanghai Key Lab. of D&A for Metal-Functional Materials, School of Materials Science & Engineering, Tongji University, Shanghai 201804, P. R. China

[2]School of Materials Science & Engineering, University of Shanghai for Science and Technology, Shanghai 200092, P. R. China

*Corresponding author. E-mail: weilu@tongji.edu.cn (Wei Lu)

Supplementary Tables and Figures

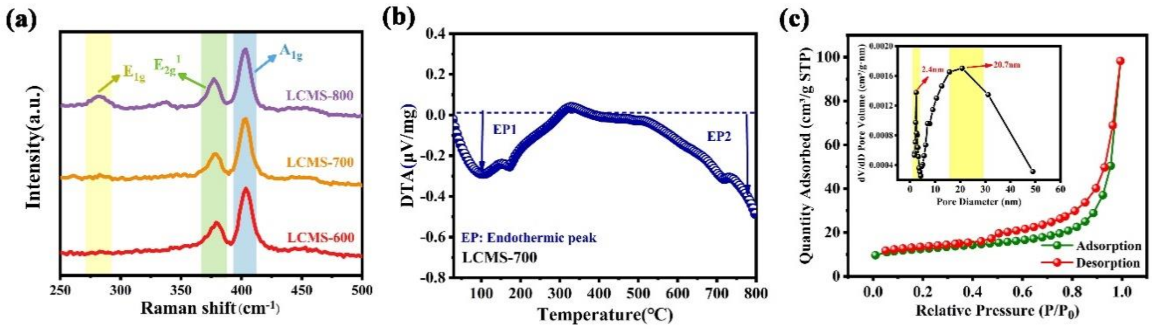

Fig. S1 **a** Raman spectroscopy of samples. **b** DTA curves of LCMS-700. **c** N_2 adsorption-desorption isotherms and pore size distribution of LCMS-700

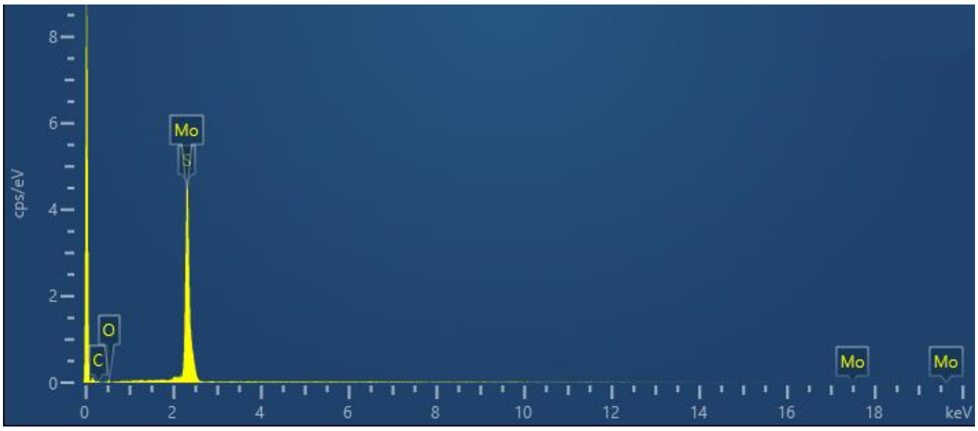

Fig. S2 Elemental mappings of S, Mo, and C elements of LCMS-700

Table S1 Elemental Mappings of LCMS-700

element	line type	wt% weight	wt% sigma	wt% atom
S	K	32.24	1.40	36.02
Mo	L	51.49	1.99	19.23
O	K	5.10	0.95	11.43
C	K	11.17	2.41	33.32
Amounts		100		100

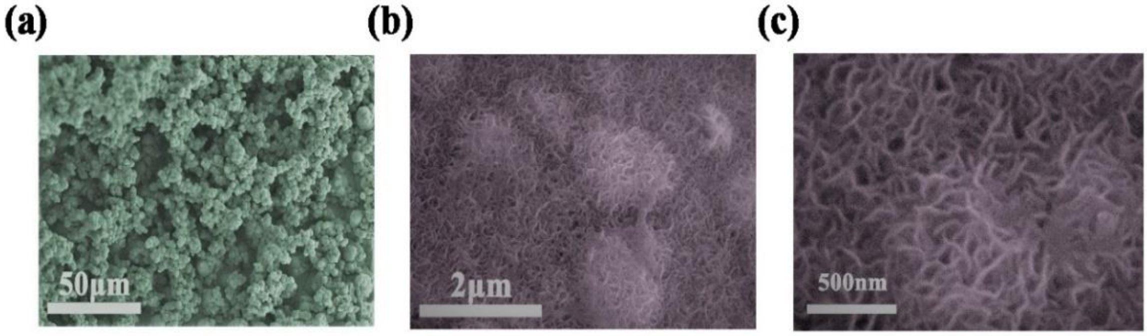

Fig. S3 a SEM images of front side of LCMS-700, **b-c** SEM images of back side of LCMS-700

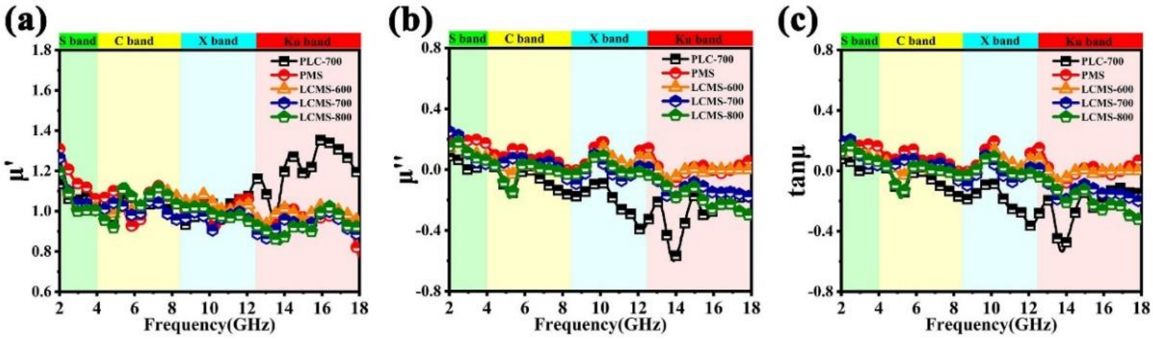

Fig. S4 EMW parameters of samples. **a** real and **b** imaginary parts of permeability. **c** magnetic loss tangents

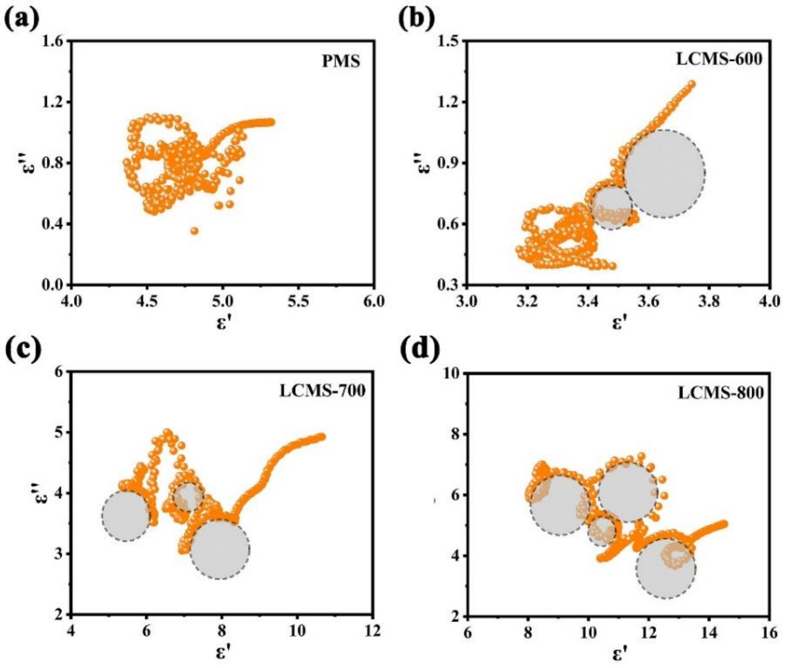

Fig. S5 Cole−Cole curves of samples: **a** PMS, **b** LCMS-600, **c** LCMS-700, **d** LCMS-800

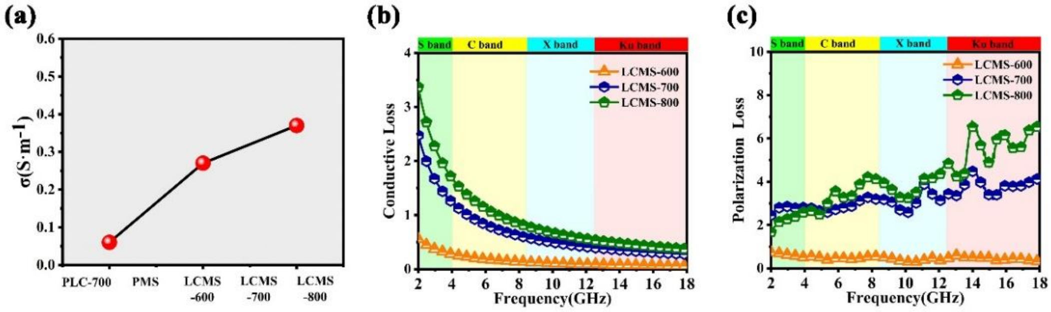

Fig. S6 a Conductivity (σ) of samples. **b-c** conduction loss (ε''_c) and polarization loss (ε''_p) of LCMS-600, LCMS-700, and LCMS-800

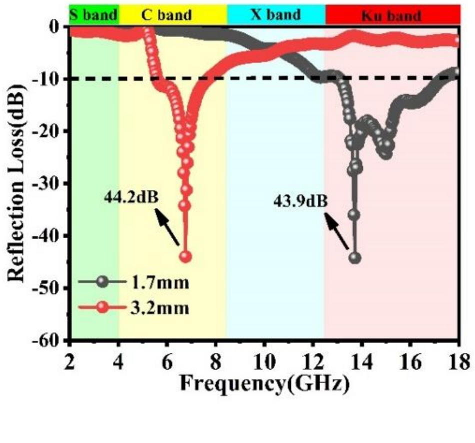

Fig. S7 Reflection loss of LCMS-800 from 2.0 to 18.0 GHz at 1.7 mm and 3.2 mm

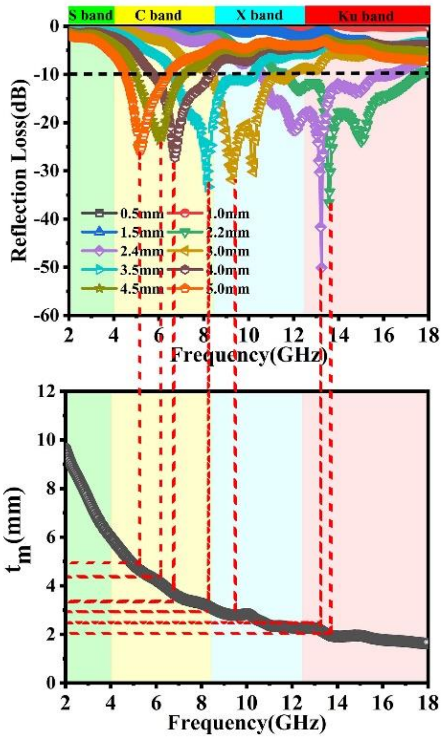

Fig. S8 Reflection loss and quarter wavelength thickness of LCMS-700 from 2.0 to 18.0 GHz

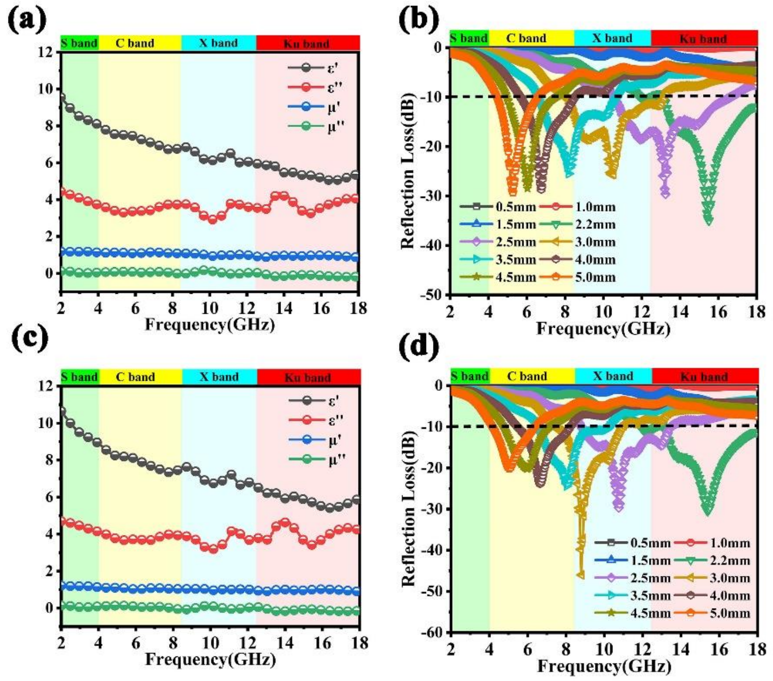

Fig. S9 EMW parameters and reflection loss at different carbonation heating rates of LCMS-700 from 2.0 to 18.0 GHz: **a-b** 3 ℃ min^{-1}; **c-d** 5 ℃ min^{-1}

Table S2 Dielectric sum-quotient model (ε_{total}=5)

ε_{total}=5	1.0 mm	1.2 mm	1.4 mm	1.6 mm	1.8 mm	2.0 mm
cotε=5.0	None	None	None	None	None	None
cotε=4.0	None	None	None	None	None	None
cotε=3.0	None	None	None	None	None	None
cotε=2.5	None	None	None	None	None	None
cotε=2.0	None	None	None	None	None	None
cotε=1.5	None	None	None	None	None	None
cotε=1.0	None	None	None	None	None	None
ε_{total}=5	2.2 mm	2.4 mm	2.6 mm	2.8 mm	3.0 mm	
cotε=5.0	None	None	None	None	None	
cotε=4.0	None	None	None	None	None	
cotε=3.0	None	16.4-18.0	15.1-17.9	14.0-16.6	13.1-15.5	
cotε=2.5	17.2-18.0	15.8-18.0	14.6-18.0	13.6-18.0	12.6-17.8	
cotε=2.0	17.0-18.0	15.6-18.0	14.4-18.0	13.3-18.0	12.4-18.0	
cotε=1.5	17.0-18.0	15.6-18.0	14.4-18.0	13.3-18.0	12.4-18.0	
cotε=1.0	17.6-18.0	16.1-18.0	14.8-18.0	13.8-18.0	12.8-18.0	

Table S3 Dielectric sum-quotient model (ε_{total}=10)

ε_{total}=10	1.0 mm	1.2 mm	1.4 mm	1.6 mm	1.8 mm	2.0 mm
cotε=5.0	None	None	None	None	None	None
cotε=4.0	None	None	None	16.2-18.0	14.4-16.4	13.0-14.8
cotε=3.0	None	None	None	15.8-18.0	14.1-18.0	12.7-16.2
cotε=2.5	None	None	None	15.8-18.0	14.1-18.0	12.6-17.0
cotε=2.0	None	None	None	16.0-18.0	14.2-18.0	12.8-17.8
cotε=1.5	None	None	None	16.4-18.0	14.6-18.0	13.1-18.0
cotε=1.0	None	None	None	None	16.1-18.0	14.4-18.0
ε_{total}=10	2.2 mm	2.4 mm	2.6 mm	2.8 mm	3.0 mm	
cotε=5.0	None	None	None	None	None	
cotε=4.0	11.8-13.4	10.8-12.3	10.0-11.4	9.2-10.5	8.6-9.8	
cotε=3.0	11.5-14.7	10.0-13.5	9.7-12.4	9.0-11.5	8.4-10.8	
cotε=2.5	11.5-15.4	10.6-14.1	9.7-13.0	9.0-12.1	8.4-11.3	
cotε=2.0	11.6-16.2	10.6-14.8	9.8-13.7	9.1-12.7	8.5-11.9	
cotε=1.5	12.0-16.9	11.0-15.5	10.1-14.3	9.4-13.3	8.8-12.4	
cotε=1.0	13.2-16.6	12.0-15.2	11.1-14.0	10.3-13.0	9.6-12.1	

Table S4 Dielectric sum-quotient model (ε_{total}=15)

ε_{total}=15	1.0 mm	1.2 mm	1.4 mm	1.6 mm	1.8 mm	2.0 mm
cotε=5.0	None	17.3-18.0	14.8-16.3	13.0-14.3	11.5-12.7	10.4-11.4
cotε=4.0	None	17.0-18.0	14.6-17.3	12.8-15.2	11.3-13.4	10.2-12.1
cotε=3.0	None	17.0-18.0	14.6-18.0	12.7-16.2	11.3-14.4	10.2-12.9
cotε=2.5	None	17.1-18.0	14.7-18.0	12.8-16.7	11.4-14.8	10.3-13.4

cotε=2.0	None	17.5-18.0	15.0-18.0	13.1-17.3	11.6-15.4	15.5-13.8
cotε=1.5	None	None	15.7-18.0	13.8-17.5	12.2-15.6	11.0-14.0
cotε=1.0	None	None	None	None	None	None
ε_{total}=15	2.2 mm	2.4 mm	2.6 mm	2.8 mm	3.0 mm	
cotε=5.0	9.4-10.4	8.6-9.5	8.0-8.8	7.4-8.1	6.9-7.4	
cotε=4.0	9.3-11.0	8.5-10.1	7.8-9.3	7.3-8.6	6.8-8.0	
cotε=3.0	9.2-11.7	8.5-10.8	7.8-9.9	7.3-9.2	6.8-8.6	
cotε=2.5	9.3-12.1	8.6-11.1	7.9-10.2	7.3-9.5	6.8-8.9	
cotε=2.0	9.5-12.6	8.7-11.5	8.0-10.6	7.5-9.8	7.0-9.2	
cotε=1.5	10.0-12.7	9.2-11.7	8.5-10.8	7.8-10.0	7.3-9.3	
cotε=1.0	None	None	None	None	None	

Table S5 Dielectric sum-quotient model (ε_{total}=20)

ε_{total}=20	1.0 mm	1.2 mm	1.4 mm	1.6 mm	1.8 mm	2.0 mm
cotε=5.0	17.6-18.0	14.6-16.6	12.5-14.2	11.0-12.4	9.8-11.1	8.8-10.0
cotε=4.0	17.5-18.0	14.6-17.4	12.5-14.9	10.9-13.0	9.7-11.6	8.7-10.4
cotε=3.0	17.6-18.0	14.7-18.0	12.6-15.7	11.0-13.7	9.8-12.2	8.8-11.0
cotε=2.5	17.9-18.0	14.9-18.0	12.8-16.1	11.2-14.1	9.9-12.5	8.9-11.2
cotε=2.0	None	15.3-18.0	13.1-16.4	11.5-14.4	10.0-12.8	9.2-11.5
cotε=1.5	None	16.6-18.0	14.2-16.2	12.4-14.2	11.0-12.6	9.9-11.3
cotε=1.0	None	None	None	None	None	None
ε_{total}=20	2.2 mm	2.4 mm	2.6 mm	2.8 mm	3.0 mm	
cotε=5.0	8.0-9.0	7.3-8.3	6.7-7.6	6.2-7.1	5.8-6.6	
cotε=4.0	7.9-9.5	7.3-8.7	6.7-8.0	6.2-7.4	5.8-6.9	
cotε=3.0	8.0-10.0	7.3-9.1	6.8-8.4	6.3-7.8	5.9-7.3	
cotε=2.5	8.1-10.2	7.4-9.4	6.9-8.6	6.4-8.0	6.0-7.5	
cotε=2.0	8.4-10.4	7.6-9.6	7.0-8.8	6.6-8.2	6.1-7.6	
cotε=1.5	9.0-10.3	8.3-9.4	7.6-8.7	7.1-8.0	6.6-7.5	
cotε=1.0	None	None	None	None	None	

Table S6 EMW Absorption Performance of MGM Materials

sample	RL (dB)	thickness (mm)	bandwidth (GHz)	Refs.
C/Fe_3O_4	22.1	2.0	4.4	[49]
C/Ni	42.2	2.0	2.3	[50]
C foam	43.6	4.7	3.3	[51]
C/$CoFe_2O_4$	49.6	2.5	3.0	[52]
SiC	48.0	3.0	1.8	[53]
C aerogels	55.0	3.5	3.6	[54]
Porous C	43.8	3.0	3.2	[55]
C/Fe	57.6	3.9	5.2	[56]
C/CNT/Co	53.5	2.9	2.4	[57]
C/ Fe_3O_4/PANI	44.8	2.7	4.7	[58]
C/MoS_2	50.1	2.4	5.8	**this work**
